# Tumor-targeted aptamer-conjugated engineered bacteria for CXCL9 cytokine delivery in non-small cell lung cancer immunotherapy

**DOI:** 10.1186/s12967-026-08194-y

**Published:** 2026-04-27

**Authors:** Qinghao Gu, Runbang Wang, Lixia Zhang, Di Pan, Chaorong Bian, Erteng Jia, Junda Chang, Hao Zhang

**Affiliations:** 1 Jiangsu Key Laboratory of Digital Intelligence in Chronic Disease Prevention and Treatment, Thoracic Surgery Laboratory, Xuzhou Medical University, Xuzhou, China; 2 Department of Thoracic Surgery, Affiliated Hospital of Xuzhou Medical University, Xuzhou, China; 3 The First Clinical Medical College of Nanjing Medical University, Nanjing, China; 4 Department of Otolaryngology, Head and Neck Surgery, The Second Affiliated Hospital of Xuzhou Medical University, General Hospital of Xuzhou Mining Group, Xuzhou, China

**Keywords:** Aptamer, Engineered bacteria, Tumor microenvironment

## Abstract

**Background:**

Bacterial cancer therapies have regained attention as a strategy to remodel the immunosuppressive tumor microenvironment (TME). Engineered bacteria equipped with tumor-targeting moieties can enhance intratumoral specificity; however, safety concerns and the need for repeat dosing limit their translational potential.

**Methods:**

Here, we developed an aptamer-conjugated engineered bacterial strain (ApCB) carrying CXCL9, and evaluated its tumor-targeting colonization and immunomodulatory effects in a subcutaneous LLC tumor model. After determining the in vitro minimum inhibitory concentration (MIC) of kanamycin and extrapolating an equivalent in vivo dose based on mouse blood volume, we implemented a tail-vein antibiotic administration strategy to precisely regulate intratumoral bacterial burden.

**Results:**

Antibiotic treatment substantially lowered peak bacterial abundance in tumors while retaining a viable intratumoral bacterial reservoir, allowing sustained bacterial proliferation and periodic CXCL9 release without repeated re-administration of engineered bacteria. In vivo, ApCB–CXCL9 treatment significantly inhibited tumor growth, induced extensive tumor necrosis, decreased Ki67 expression, and increased intratumoral CD8⁺ T-cell infiltration together with elevated effector cytokines (IFN-γ, TNF-α).

**Conclusion:**

These findings indicate that aptamer-guided engineered bacteria combined with antibiotic-mediated population control can safely and controllably remodel the tumor immune microenvironment, offering a practicable approach for sustained delivery and clinical translation of bacterial immunotherapies.

**Supplementary Information:**

The online version contains supplementary material available at 10.1186/s12967-026-08194-y.

## Introduction

Cancer immunotherapy, particularly intervention systems represented by immune checkpoint blockade (ICB), has significantly improved patient outcomes for multiple solid tumors in recent years. However, extensive clinical data indicate that over half of patients exhibit limited response to ICB; in most solid tumors [1], the objective response rate (ORR) typically ranges between 10% and 30% [2, 3]. For instance, the ORR for PD-1 inhibitors in melanoma is 33%–36%, while in non-small cell lung cancer (NSCLC) and renal cell carcinoma (RCC) it is merely 41% and 21% respectively [4]. Multiple factors collectively diminish ICI efficacy, including the pervasive presence of immunosuppressive cells within the tumor microenvironment (TME), T-cell effector dysfunction, tumor metabolic reprogramming [5], and immune evasion mediated by driver mutations and multiple signal pathways (such as β-catenin, MYC, PTEN, etc.) [6–9]. Consequently, overcoming these limitations to enhance ICI response rates remains a central challenge in contemporary tumor immunology research.

Among numerous limiting factors, T cell exhaustion is widely recognized as a key bottleneck in the failure of immune checkpoint blockade (ICB) therapies [10, 11]. Sustained antigenic stimulation leads to diminished effector function in CD8⁺ T cells, manifested by significant upregulation of inhibitory receptors (such as PD-1, TIM-3, LAG-3) [12] and reduced production of effector cytokines (such as IFN-γ, TNF). Recent studies reveal that exhausted T cells exhibit distinct lineage differentiation characteristics, including proliferative precursor exhausted T cells (Tpex) with moderate PD-1 expression and terminally exhausted T cells (Tex) exhibiting high inhibitory activity and functional exhaustion [13, 14]. The dynamic equilibrium between these subpopulations profoundly influences therapeutic sensitivity to immune checkpoint blockade (ICB) [15], suggesting that enhancing intratumoral T cell recruitment and functional status represents a crucial strategy for improving ICB efficacy [16].

Concurrently, the interaction between microorganisms and the host immune system is increasingly recognized as a crucial regulator of cancer immunity [17]. Engineered bacteria have garnered significant attention due to their programmability and local delivery capabilities [18]. These engineered organisms can selectively colonize the hypoxic, nutritionally constrained environment inherent to tumors [19]. Through endogenous or induced expression of diverse immunomodulatory molecules, they reshape the local immune landscape [20]. Advances in synthetic biology have endowed them with the capacity for spatiotemporally controlled expression upon specific stimuli [21] (such as lactate, hypoxia, or metabolic signals), positioning them as potential tools for precise immune modulation. Existing studies demonstrate that engineered bacteria can regulate checkpoint molecule expression, enhance T-cell infiltration, and alter the immune composition of the tumor microenvironment (TME), offering potential pathways to alleviate T-cell exhaustion [22].

The pathological characteristics of the tumor microenvironment provide a unique ecological niche for engineered bacteria to colonize. Dysregulated tumor angiogenesis leads to widespread hypoxia [23], while tumor cells’ preference for glycolytic pathways further causes lactic acid accumulation [24] and metabolic disruption, resulting in a TME characterized by hypoxic, hyper-lactic, and immunosuppressive conditions [25, 26]. Current evidence lacks confirmation of a stable “native microbiota” within tumor tissues [27], thereby facilitating the ecological advantage of exogenous engineered bacteria within the tumor microenvironment. Previous studies indicate that attenuated bacteria can modulate multiple immune cell types, including CD4⁺ T cells, CD8⁺ T cells, Tregs, and tumor-associated macrophages (TAMs), thereby enhancing antitumor immunity [28].

Despite these advantages, engineered bacteria remain susceptible to host immune surveillance within the systemic circulation, leading to their premature clearance before reaching tumors [29]. Concurrently, the inevitable exposure of bacteria to multiple organs during circulation may pose additional risks to immunocompromised individuals. Consequently, enhancing the ability of engineered bacteria to target tumors represents a critical step in improving therapeutic efficacy while mitigating systemic risks [30].

Aptamers have emerged as crucial tools for targeted drug delivery and surface modification of nanomaterials due to their high affinity, specificity, structural programmability, and low immunogenicity [31, 32]. Through chemical modification at the 5′ or 3′ ends, aptamers can achieve stable conjugation with carriers and acquire precise cell recognition capabilities [33]. Previous literature has documented the ability of certain aptamers to specifically bind solid tumor cells; however, their targeting efficacy within mouse lung adenocarcinoma models remains unvalidated [30]. This study employs such aptamers to engineer bacterial surface modifications, thereby enhancing their targeted colonization capacity within lung adenocarcinoma. This approach expands the application scope of aptamers within tumor delivery systems [30].

**Fig. 1:**
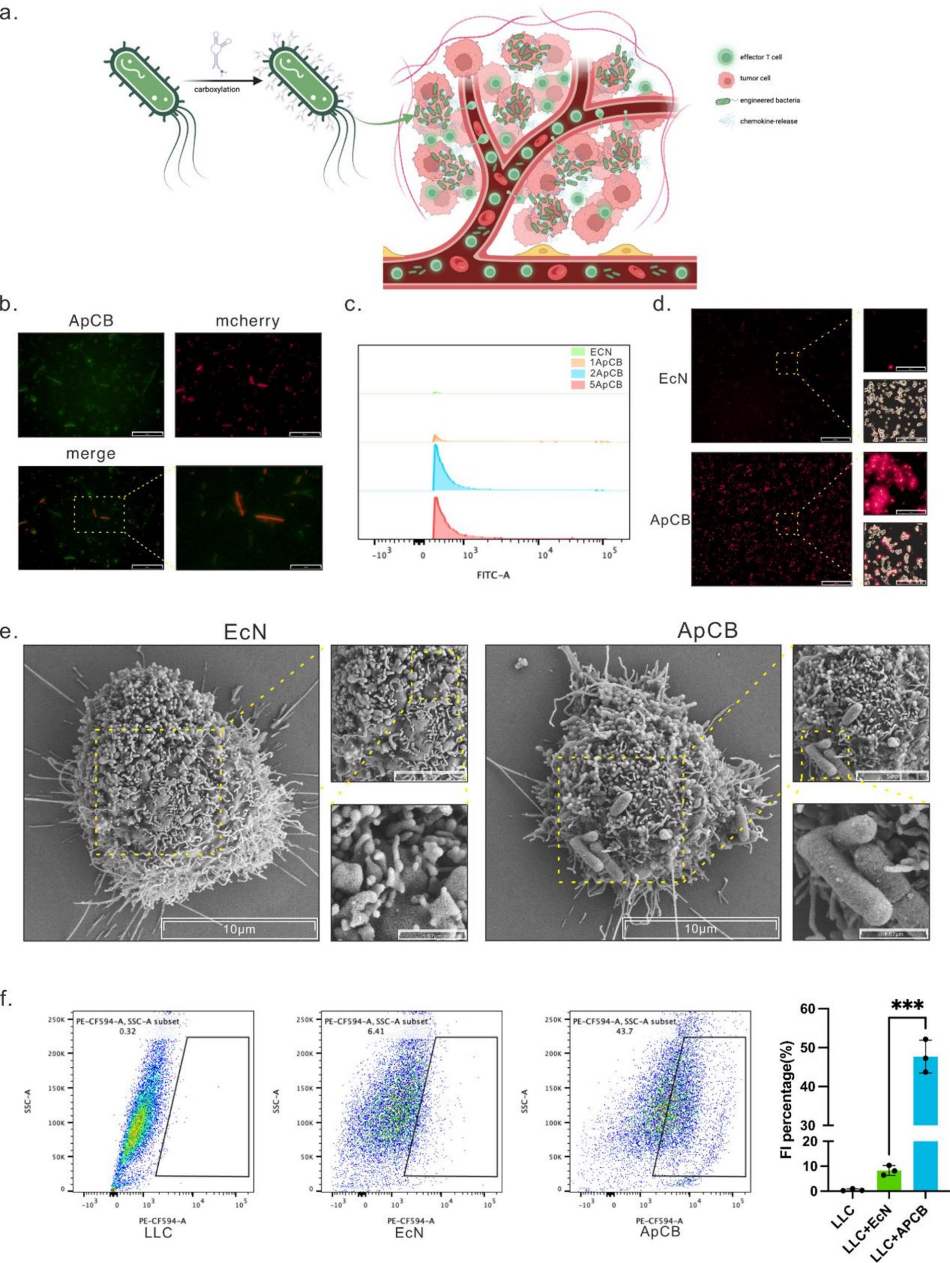
Design, preparation and characterization of ApCB. (**a**) Schematic representation of APCB: AS1411 conjugated with EcN. The process by which APCB enters the tumor from the bloodstream. Created with BioRender.com. (**b**) Laser scanning confocal microscopy images of aptamer-bound bacteria. Aptamer-labelled bacteria are visualized under microscopy. The green channel displays 6-FAM-labelled aptamers, while the red channel shows EcN-labelled aptamers expressing the mecherry protein. The figure presents representative results from three independent experiments. Biological samples. Scale bars: 50 μm and 10 μm respectively. (**c**) Flow cytometric analysis of fluorescence intensity for 6-FAM and mcherry Fluorescence after incubating EcN_mecherry_ with 6-FAM-labelled AS1411 at concentrations of 1 nmol/1 × 10⁷ CFU, 2 nmol/1 × 10⁷ CFU, and 5 nmol/1 × 10⁷ CFU for 3 h. (**d**) The figure shows laser scanning microscope images of LLC cells co-treated with EcN_mecherry_ or 5ApCB_mecherry_, incubated at 37 °C for 3 h. The images represent results from three independent biological replicates. Scale bars indicate 200 μm and 50 μm respectively. (**e**) Scanning electron microscopy images of LLC cells co-treated with EcN_mecherry_ or 5ApCB_mecherry_ and incubated at 37 °C for 3 h. Representative results from three independent biological replicates are shown. Scale bars indicate 10 μm, 5 μm, and 1.67 μm respectively. (**f**) The figure shows flow cytometric analysis of LLC cells following co-treatment with EcN_mecherry_ or 5ApCB_mecherry_, incubated at 37 °C for 3 h. Data are presented as mean ± SD. Significance was assessed using Student’s t-test (two-tailed), yielding a p-value of 0.0001 for the comparison between the 5ApCBmecherry-treated group and the EcN_mecherry_-treated group. ns: no significant difference

Against this backdrop, the present study designed an aptamer-modified engineered bacterial platform. Following in vivo administration, the engineered bacteria enter the bloodstream and achieve specific targeting and colonization of tumor cells via surface nucleic acid aptamers. Within the tumor, the engineered bacteria continuously express and release the chemokine CXCL9, forming a local chemotactic gradient that attracts CD8⁺ T cells to infiltrate the tumor and ameliorates T cell exhaustion (Fig. 1a). By integrating “aptamer targeting, engineered bacterium colonization, and chemokine delivery” within a single platform, this study aims to enhance the tumor immune microenvironment, offering a programmable therapeutic strategy to overcome the limited response to immune checkpoint inhibitor therapies.

## Results

### Characterization of the aptamer-conjugated engineered bacterial system

AS1411 is a nucleic acid aptamer capable of specifically recognizing and binding to nucleolin, which is highly expressed on the surface of tumor cells [34, 35]. It has demonstrated excellent tumor targeting capabilities in models such as breast cancer (4T1) and liver cancer (H22) [30]. However, its applicability in lung adenocarcinoma models remains unvalidated. This study aims to evaluate the targeting efficacy of AS1411 within the Lewis lung carcinoma (LLC) cell line, thereby expanding the application spectrum of this aptamer and laying the groundwork for its potential use in lung cancer immunotherapy.

To enable directed modification of bacteria by the aptamer, we introduced an amino group at the 3’-end of AS1411 for convenient conjugation, while labelling the 5’-end with 6-FAM for fluorescent detection. The engineered bacterial chassis selected was E. coli Nissle 1917 (EcN), renowned for its excellent safety profile. Bacterial surface carboxyl groups were activated via an EDC/NHS system) [30], enabling covalent amide bond formation to co-ligate the amino-modified aptamer onto the EcN surface (Fig. 1a). First, we observed the conjugation efficiency under fluorescence microscopy: mCherry-expressing EcN served as the red signal source, while 6-FAM conferred green fluorescence to the aptamer. Merged channel images revealed high overlap between green and red signals, indicating stable aptamer modification on the engineered bacterial surface (Fig. 1b).

Subsequently, we employed flow cytometry to further quantify the aptamer conjugation efficiency. As the AS1411 concentration in the reaction system increased, the green fluorescence intensity of the bacteria exhibited a dose-dependent rise, confirming that the degree of aptamer modification could be controllably adjusted via the feed ratio (Fig. 1c). Thereby, we use 5 nmol/1 × 10^7^CFU in our subsequent experiments.

To validate the tumor-binding capacity of the modified engineered bacteria, we introduced both aptamer-modified and unmodified EcN strains into an in vitro co-culture system containing LLC cells at equal densities. Fluorescence imaging revealed that unmodified EcNs exhibited only scattered presence on the LLC cell surface, whereas AS1411-modified engineered bacteria significantly enriched around tumor cells, demonstrating markedly enhanced binding capacity (Fig. 1d). Scanning electron microscopy further confirmed this finding: compared to unmodified bacteria, AS1411-modified EcN exhibited a higher level of attachment to the surface of LLC cells (Fig. 1e).

Finally, we quantitatively analyzed the binding efficiency of engineered bacteria to LLC cells via flow cytometry. Results demonstrated that over 40% of LLC cells exhibited detectable fluorescent signals from engineered bacteria on their surfaces, further supporting that AS1411 modification significantly enhances the binding probability between engineered bacteria and lung cancer cells (Fig. 1f).

Collectively, these findings demonstrate that the AS1411 aptamer successfully and efficiently modifies engineered bacteria while enhancing their binding capacity to lung adenocarcinoma cells, providing a robust foundation for advancing tumor-specific delivery strategies using engineered bacteria.

### CXCL9 can recruit T cells into the tumor microenvironment both in vitro and in vivo

CXCL9 belongs to the CXC chemokine family and is typically upregulated in innate immune cells such as macrophages and dendritic cells following stimulation by inflammatory mediators [36]. It primarily exerts its function by binding to the receptor CXCR3, which is widely expressed on effector CD8⁺ T cells, CD4⁺ T cells, and natural killer (NK) cells [37]. Together with CXCL10 and CXCL11, CXCL9 constitutes the CXCR3 signal axis, driving the directed migration of cytotoxic lymphocytes [36] towards tumors or sites of inflammation and influencing their spatial distribution within the tumor microenvironment. Previous studies indicate this axis plays a crucial role in enhancing T-cell infiltration in “immunologically cold” tumors and boosting responses to immune checkpoint therapy [38]. T-cell exhaustion, however, represents a key mechanism underpinning late-stage resistance and non-response to immunotherapy [39]. Consequently, we aim to employ engineered bacteria for sustained local release of CXCL9 within tumors, thereby enhancing the infiltration of anti-tumor immune cells and improving immunotherapy efficacy [40].

**Fig. 2:**
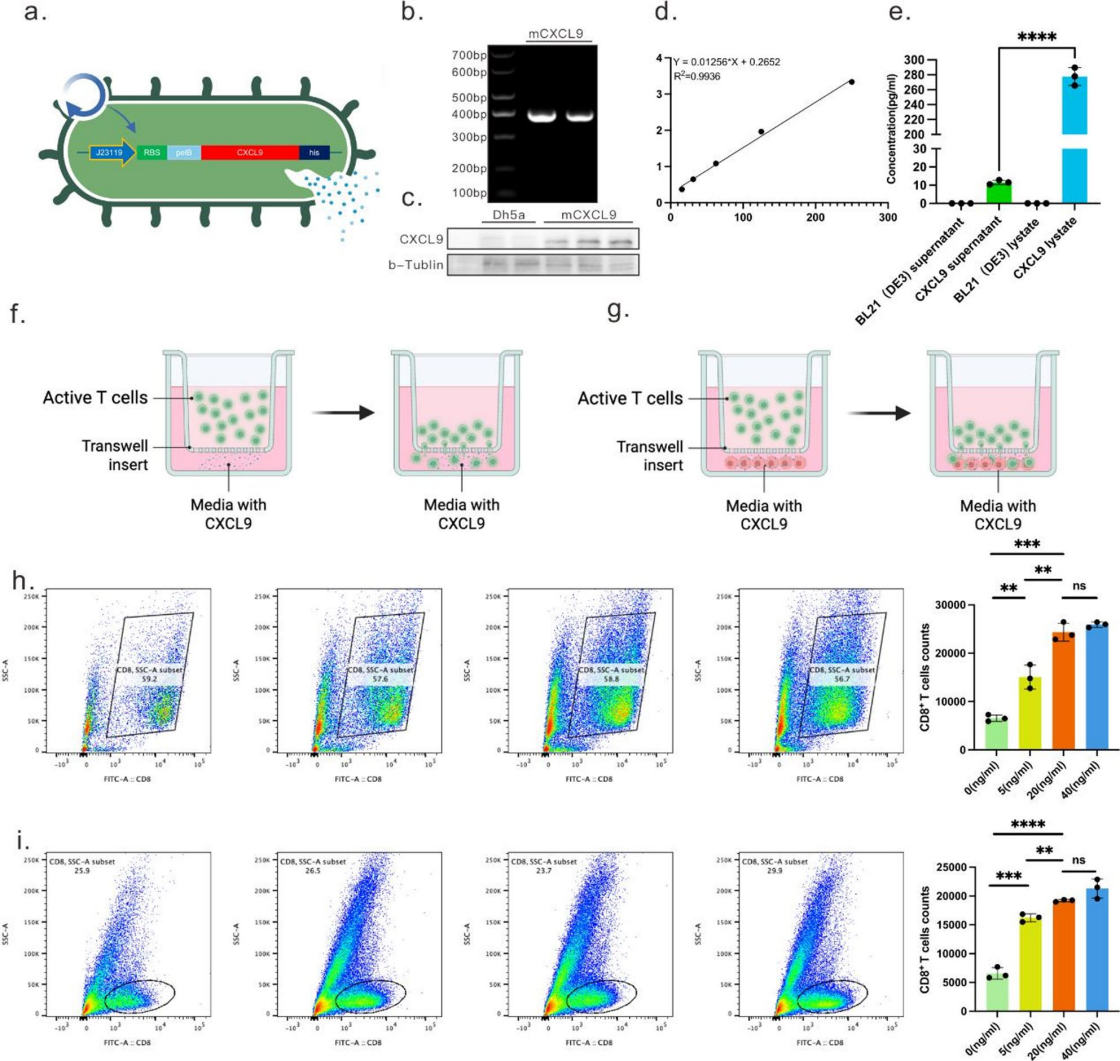
Functional Validation of CXCL9 Production by Engineered Bacteria. (**a**) Schematic diagram of the plasmid in EcN_CXCL9_, showing the modified region of the genome. Created with BioRender.com. (**b**) Gel electrophoresis imaging of PCR from EcN_CXCL9_ bacterial culture. (**c**) Protein expression levels of CXCL9 in EcN_CXCL9_ determined by Western blotting. (**d**) CXCL9 concentrations were calculated using linear regression of the ELISA standard curve. (**e**) Detection of CXCL9 protein activity in EcN_CXCL9_ via ELISA. f-i. Single-cell transwell assay: Upper chamber loaded with CD3^+^ T cells (1 × 10⁶ cells/well) derived from C57BL/6J mouse spleen; lower chamber loaded with purified CXCL9 protein and/or LLC cells (1 × 10⁵ cells/well) for inter-chamber migration (**f**-**g**). After 24 h of incubation, cells migrating into the lower chamber were collected and stained with fluorescently labelled antibodies against CD3 and CD8. Flow cytometric analysis was performed to identify CD8^+^ T cells within the CD3^+^ T cell population (**h**-**i**)

To achieve efficient expression of CXCL9 in engineered bacteria, we fused the pelB signal peptide to the N-terminus of CXCL9 to enhance its solubility under overexpression conditions and facilitate secretion into the periplasm (Fig. 2a). We first validated CXCL9 expression in engineered bacteria via DNA gel electrophoresis (Fig. 2b). Through Western blot and ELISA analysis of engineered bacterial lysates, we confirmed the successful expression of CXCL9 and the integrity of its secreted form (Fig. 2c-e). Subsequently, we evaluated the biological activity of CXCL9 derived from the engineered bacteria. First, its in vitro recruitment effect was assessed using a Transwell system. Activated T cells were added to the upper chamber, while purified CXCL9 protein was added to the lower chamber (Fig. 2f). Flow cytometry revealed that CXCL9 significantly promoted activated T-cell migration, with the number of migrating cells increasing in a dose-dependent manner with rising CXCL9 concentrations (Fig. 2h). Among T cells migrating to the lower chamber, the proportion of CD8⁺ T cells remained approximately 60%, indicating that the expressed CXCL9 exhibits stable chemotactic directionality. To further simulate the scenario of engineered bacteria locally releasing CXCL9 within the tumor microenvironment, we added LLC tumor cells to the lower chamber of the Transwell system and co-administered CXCL9 (Fig. 2g). Results demonstrated that activated T cells were similarly recruited to the subchamber, exhibiting migration patterns consistent with CXCL9 treatment alone (Fig. 2i), indicating that CXCL9 retains chemotactic activity even within tumor microenvironments.

To exclude the possibility that tumor cells may secrete endogenous chemokines that confound the observed CXCL9-mediated chemotactic effects, we included tumor cell-conditioned medium (TCM) as an additional control. No significant difference in T cell recruitment was observed between the TCM group and the blank medium control (Supplementary Fig. 1a–b), indicating that tumor-derived soluble factors alone do not induce detectable chemotactic activity under our experimental conditions. Furthermore, we assessed CXCR3 expression on the recruited CD8⁺ T cells. Although the absolute number of migrated cells varied among groups, flow cytometry analysis revealed comparable CXCR3 expression levels across all conditions, including the TCM and CXCL9-treated groups (Supplementary Fig. 1c–d). These results suggest that the enhanced T cell recruitment is primarily driven by exogenously added CXCL9 rather than tumor cell-derived factors and is not associated with alterations in CXCR3 expression.

**Fig. 3:**
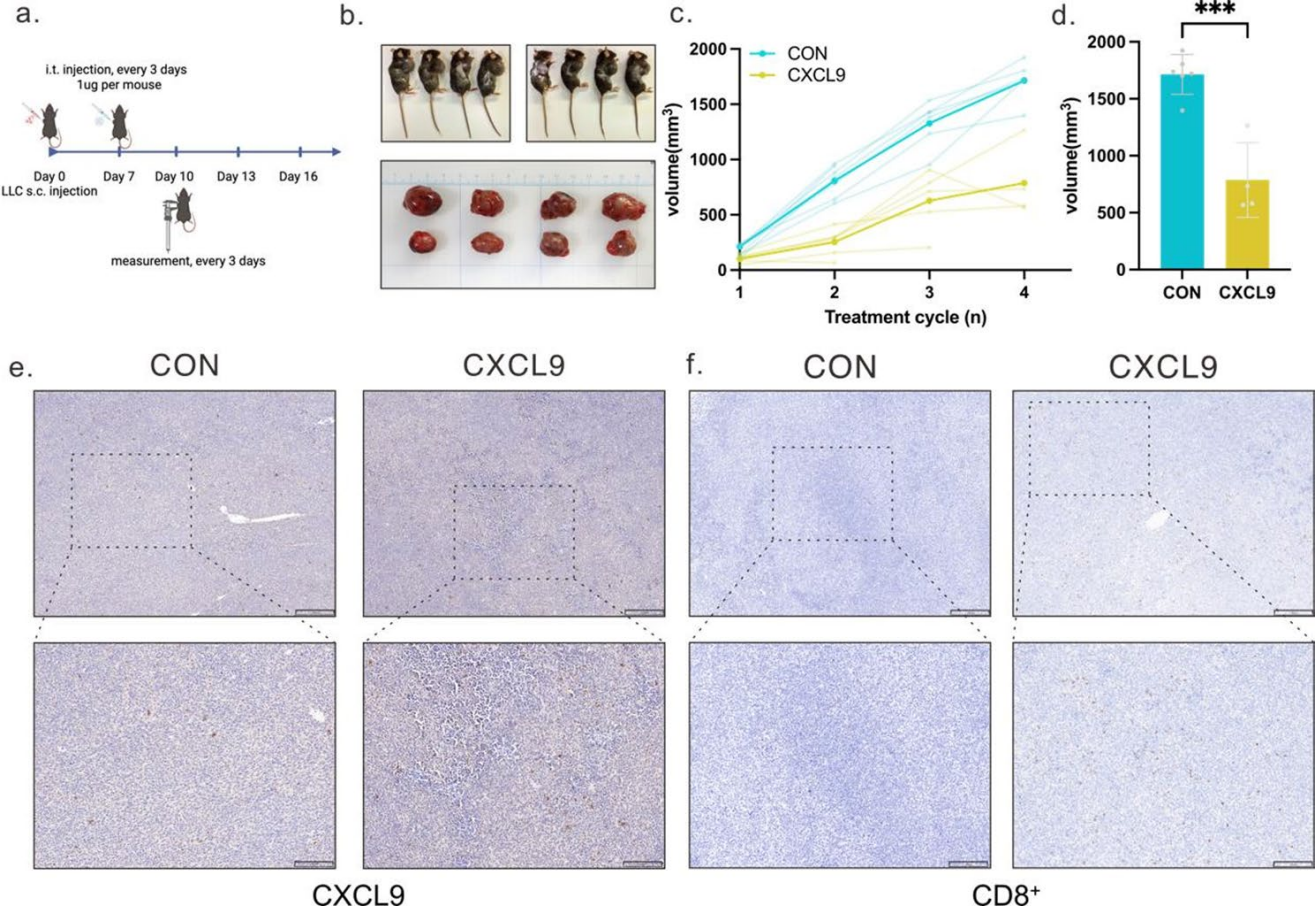
In Vivo Functional Validation of CXCL9. (**a**) Schematic diagram of intratumoral injection administration in mice. Created with BioRender.com. (**b**) Photograph of tumor tissue extracted from mice at the end of treatment. (**c**) Individual growth curves and average tumor growth curve for LLC tumors. (**d**) Relative tumor growth in the control group versus the CXCL9-treated group (*n* = 4). Data are presented as mean ± SD. Significance was assessed using Student’s t-test (two-tailed), yielding a p-value of 0.0004 between the CXCL9 and control groups. ns: non-significant. (**e**) Immunohistochemical staining images of CXCL9 in tumor tissue sections from the control and CXCL9-treated groups. (**f**) Immunohistochemical staining images of CD8^+^ in tumor tissue sections from the control and CXCL9-treated groups

Following validation of in vitro function, we established a subcutaneous LLC tumor model in mice to assess CXCL9’s immune recruitment efficacy in solid tumors (Fig. 3a). Intratumoral injection of purified CXCL9 at a dose of 40 µg/kg significantly inhibited tumor growth in mice (Fig. 3b-d). Immunohistochemical analysis revealed markedly elevated CXCL9 expression within tumors (Fig. 3e) and increased CD8⁺ T cell density (Fig. 3f) in the CXCL9-injected group compared to controls, further demonstrating CXCL9’s efficacy in promoting T cell infiltration in vivo.

To further determine whether the recruited CD8⁺ T cells exhibit functional cytotoxic potential in vivo, we performed immunofluorescence staining on tumor sections. Consistent with our previous immunohistochemical findings, CXCL9 treatment markedly increased CD8⁺ T cell infiltration within the tumor microenvironment (Supplementary Fig. 2a). We next assessed the expression of cytotoxicity-associated markers, interferon-γ (IFN-γ) and CD107b, by immunofluorescence staining. Compared with the control group, tumors from CXCL9-treated mice displayed enhanced expression of IFN-γ and CD107b within CD8⁺ T cell–enriched regions (Supplementary Fig. 2b). These findings indicate that the recruited CD8⁺ T cells are functionally active and possess cytotoxic potential, rather than representing a non-functional or exhausted phenotype.

In summary, CXCL9 engineered for systemic expression not only exhibits stable, concentration-dependent T cell recruitment activity in vitro but also enhances CD8⁺ T cell accumulation at tumor sites in vivo. This establishes a functional foundation for subsequent immunotherapy strategies involving engineered bacteria for targeted CXCL9 delivery.

### Verification of the in vivo targeted effects of aptamer-conjugated engineered bacteria

**Fig. 4:**
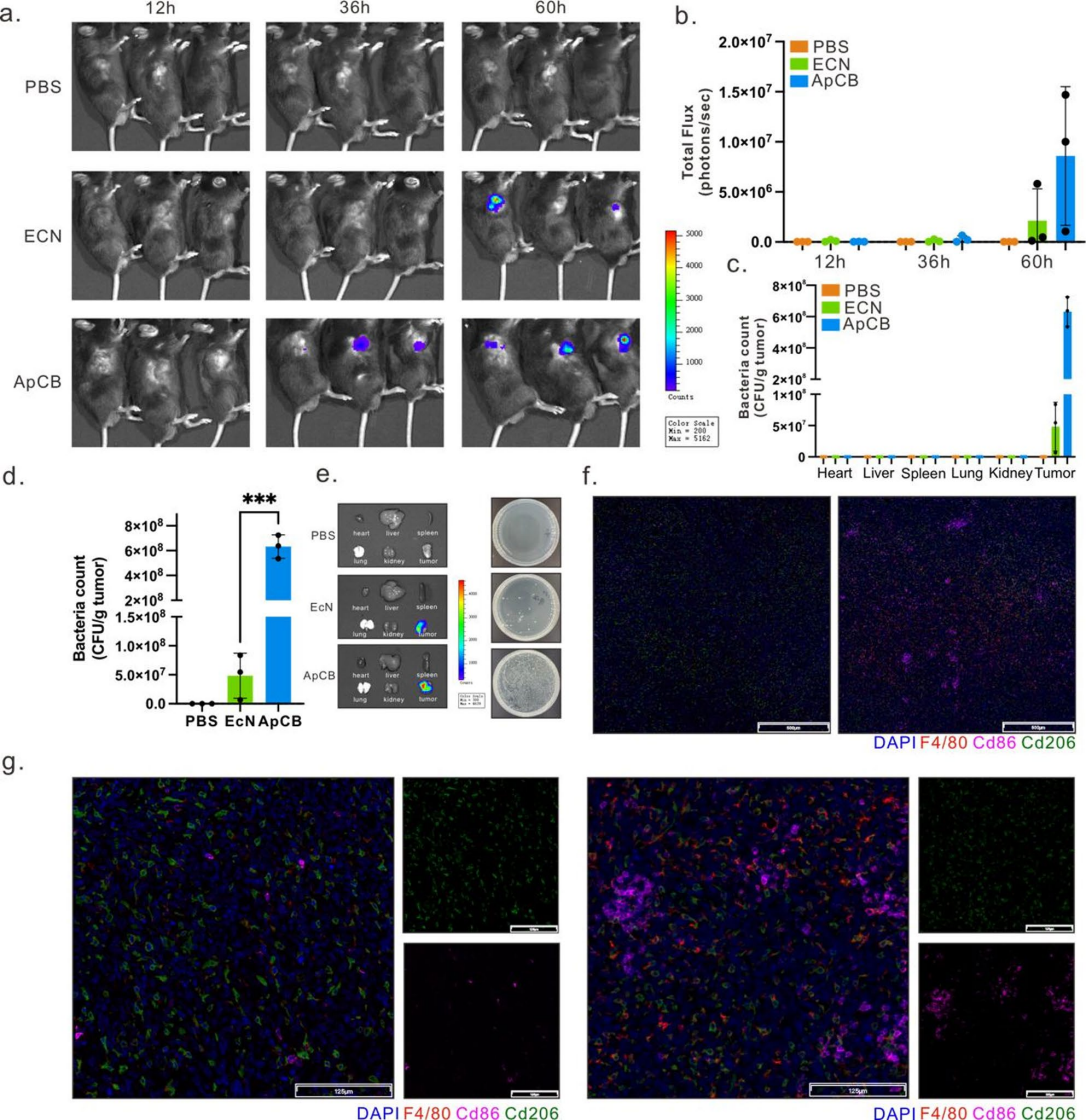
Biological distribution of ApCB in tumor-bearing mice. (**a**) IVIS imaging of tumor sites in LLC-bearing mice at 12, 36, and 60 h post-intravenous tail vein injection of PBS, 1 × 10⁷ CFU EcN_Lux_, and 5ApCB_Lux_ (*n* = 3). (**b**) Quantification of luminescence intensity (atomic units, a.u.) by delineating regions of interest encompassing the entire solid tumor at three time points (*n* = 3). **c**-**e**. In vitro IVIS Lumina III system results: mean luminescence intensity detected in tumors and major organs of LLC-bearing mice at 60 h post-tail vein injection of PBS or 1 × 10⁷ CFU EcN_Lux_ and 5ApCB_Lux_ (**e**). At the 60-hour time point, major organs and tumor tissues from each group were ground and plated. Bacterial survival rates of EcN_Lux_ and 5ApCB_Lux_ in major organs (**c**) and tumor tissues (**d**) were determined via LB agar plate counting. Plates were incubated at 37 °C for 24 h before colony counting (*n* = 3 independent experiments). Data are presented as mean ± SD. Student’s t-test (two-tailed) was used to assess significance, yielding p-value: 0.0006 for 5ApCBLux versus EcN_Lux_. ns: non-significant. f-g. Immunofluorescence staining images of tumor tissue from the PBS and 5ApCB_Lux_ groups. In the images, blue indicates DAPI-stained nuclei, red denotes F4/80-stained total macrophages, purple represents Cd86-stained M1 (classically activated) macrophages, and green shows Cd206-stained M2 (classically activated) macrophages. Scale bar: 125 μm

To validate the targeting performance of the aptamer in mice, we first conducted experiments in a subcutaneous tumor model. In the initial trial, we employed engineered bacteria carrying the mCherry fluorescent tag as a tracer to observe bacterial localization within the tumor. However, due to limitations in mCherry’s emission wavelength and signal intensity, its distribution could not be clearly visualized in the in vivo imaging system. Therefore, to obtain more intuitive and continuous bacterial localization information at the in vivo level, we further employed a luminescent bacterial strain expressing pGEN-luxCDABE, conjugating the AS1141 aptamer to the bacterial surface, whilst establishing PBS and EcN as control groups. Bacteria from different groups were administered to mice via caudal vein injection, with in vivo imaging conducted at 12 h, 36 h, and 60 h. Results demonstrated that between 12 and 60 h, the luminescence signal in the tumor region of the ApCB group progressively intensified over time, remaining significantly higher than the control groups at each time point (Fig. 4a). Notably, at 60 h, the average photon intensity in the ApCB group reached several times that of the PBS and EcN groups, further confirming the aptamer’s ability to significantly enhance the in vivo tumor enrichment capacity of engineered bacteria (Fig. 4b). To elucidate the actual distribution of engineered bacteria across different organs in vivo, mice were euthanized post-imaging. Heart, liver, spleen, lung, kidney, and tumor tissues were collected, homogenized, and plated on LB agar for culture. Colony counts revealed approximately four times more colonies in tumors from the ApCB group compared to the EcN group (Fig. 4c-d), while distribution in other organs remained limited (Fig. 4e). This indicates that the aptamer promotes a greater tendency for engineered bacteria to colonize tumor regions.

Furthermore, as a Gram-negative bacterium, the natural death of Escherichia coli within tumor regions may lead to increased release of lipopolysaccharide (LPS), and existing research indicates that LPS can induce macrophage polarization towards the M1 phenotype. Tumor-associated macrophages (TAMs) constitute a significant component of tumor-infiltrating immune cells. In most solid tumors, they predominantly exhibit an immunosuppressive M2 phenotype, representing a critical factor influencing the tumor immune microenvironment and immunotherapy response. Consequently, we further performed multiplex fluorescence staining on tumor tissues with and without engineered bacterial treatment. Results revealed a marked increase in the local accumulation of CD86⁺ (M1-like) macrophages within tumor tissue following engineered bacterium colonization (Fig. 4f–g). To further characterize macrophage polarization within the tumor microenvironment, we performed flow cytometric analysis on dissociated tumor tissues to quantify macrophage subsets. The overall proportion of M1-like macrophages (CD86⁺) was not significantly altered following bacterial treatment (Supplementary Fig. 3a, c). In contrast, the proportion of immunosuppressive M2-like macrophages (CD206⁺) was markedly reduced in the treated group compared with controls (Supplementary Fig. 3b, d), resulting in a significantly increased M1/M2 ratio (Supplementary Fig. 3e).

Notably, although no significant change in the overall proportion of M1-like macrophages was detected by flow cytometry, our immunofluorescence analysis suggests a localized increase in CD86⁺ macrophage accumulation within the tumor tissue, which may reflect differences between spatial distribution and bulk quantification approaches. Importantly, the pronounced reduction in M2-like macrophages indicates that bacterial therapy alleviates immunosuppressive macrophage polarization and contributes to remodeling of the tumor microenvironment toward a more pro-inflammatory state.

### Antibiotic-induced controlled cracking and release of engineered bacteria in tumor regions

**Fig. 5:**
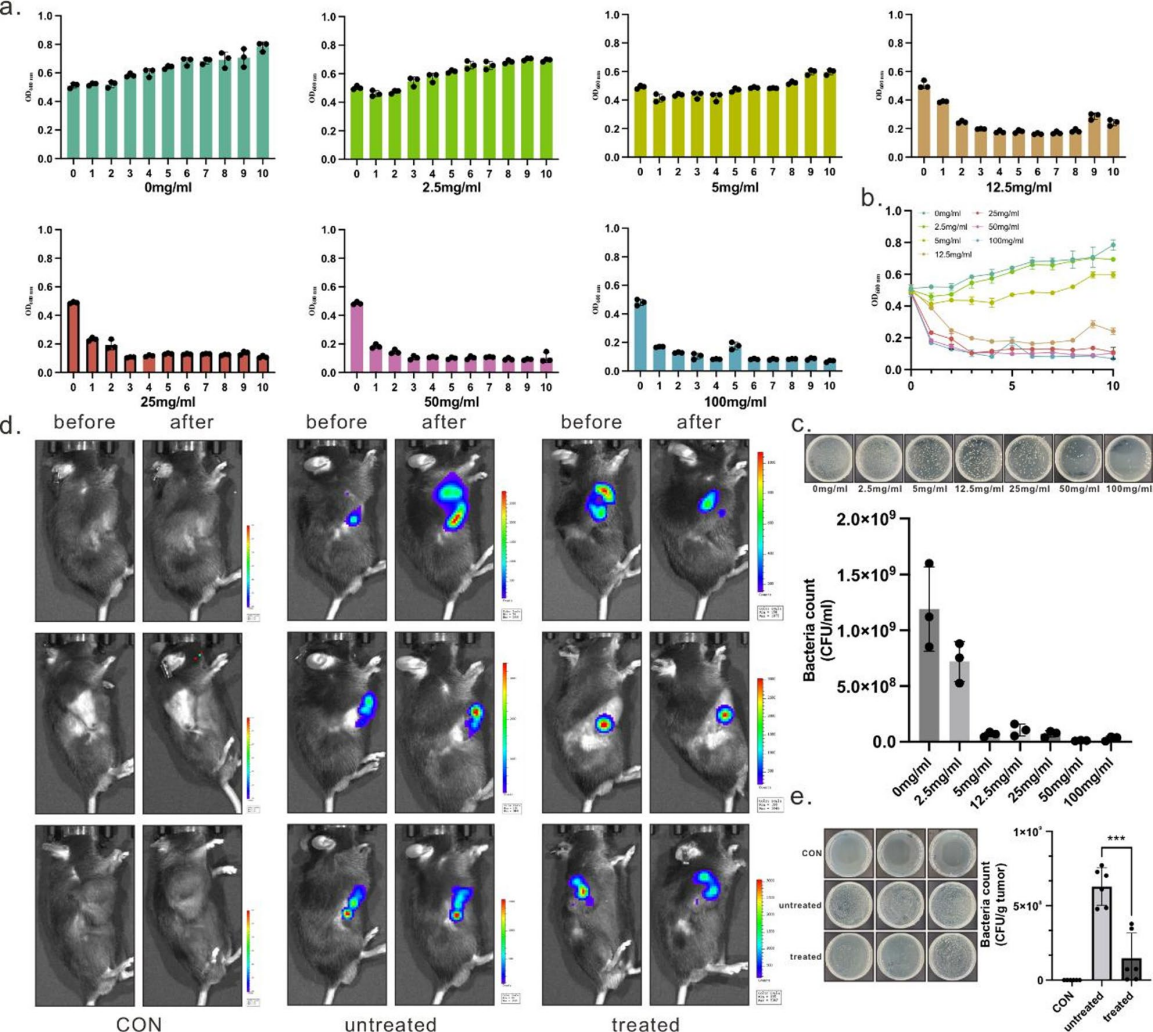
Antibiotic-Controlled Verification of Intratumoral Engineered Bacterial Activity. (**a**) Growth curves of the 5ApCB_Lux_ group and the 5ApCB_Lux_ kanamycin-treated group cultured in LB liquid medium. Kanamycin concentrations were 0 mg/ml, 2.5 mg/ml, 5 mg/ml, 12.5 mg/ml, 25 mg/ml, 50 mg/ml, and 100 mg/ml. OD600 values were recorded hourly using a microplate reader. (**b**) Growth curves of 5ApCB_Lux_ treated with varying kanamycin concentrations. (**c**) When the control group reached the growth plateau phase, bacterial suspensions at each antibiotic concentration were diluted 1000-fold at the 10-hour mark for plate spreading. Colony counts on agar plates were analyzed using ImageJ. (**d**) Treatment of LLC tumor-bearing mice (*n* = 3) with PBS, 5ApCB_Lux_, and 5ApCB_Lux_ supplemented with kanamycin. Kanamycin was administered via tail vein injection at a concentration of 1 mg/ml. Using the Caliper IVIS Lumina III system, the average luminescence signal intensity at the tumor site prior to kanamycin injection and 24 h post-injection was quantified by plotting regions of interest encompassing the entire solid tumor (*n* = 3), expressed in atomic units (a.u.). (**e**) Tumor tissue plating. Bacterial survival rates were determined for the PBS, 5ApCB_Lux_, and 5ApCB_Lux_-kanamycin-treated groups using the LB agar plate count method. Tumor tissues were homogenized and plated, and plates were incubated at 37 °C for 24 h prior to colony counting (*n* = 3). Colony counts were quantified using ImageJ. Notably, antibiotic treatment markedly reduced intratumoral bacterial burden compared with the untreated group, although residual viable bacteria remained detectable. Data are presented as mean ± SD. Statistical significance was determined using a two-tailed Student’s t-test (ns, not significant)

To determine the antibiotic dosage suitable for regulating the lysis of engineered bacteria in vivo, we first conducted in vitro kanamycin sensitivity testing on the engineered bacteria. In engineered bacterial cultures at the logarithmic phase (OD600 ≈ 0.5) (total volume 5 mL), 5 µL of kanamycin stock solution at concentrations ranging from 0 to 100 mg/mL was added, resulting in final drug concentrations ranging from 0 to 100 µg/mL (Fig. 5a). The inhibition curves revealed that bacterial proliferation began to be suppressed at a final drug concentration of approximately 5 µg/mL. Significant inhibition occurred at 12.5 mg/mL (Fig. 5a-c), accompanied by observable cell debris precipitation, indicating this concentration approaches the in vitro minimum inhibitory concentration (MIC) for the engineered bacteria.

Based on the in vitro MIC, we estimated the antibiotic exposure in mice by converting to blood volume equivalents. A 25 g mouse has a blood volume of approximately 1.75 mL. To achieve a plasma peak concentration equivalent to the in vitro MIC (12.5 µg/mL) in vivo, approximately 21.9 µg of kanamycin would be required. To account for in vivo distribution, clearance, and tissue penetration, we employed 5–10 times the in vitro MIC as the initial in vivo dose, equating to approximately 110–220 µg per mouse (roughly 5–9 mg/kg). Ultimately, a dose of 5 mg/kg was selected for experimentation, administered via a single tail vein injection to achieve rapid in vivo exposure. This dosage range offers controllable safety margins while theoretically enabling partial lysis of engineered bacteria, yet preserving sufficient viable populations for subsequent repopulation.

Subsequently, we administered antibiotics via tail vein injection into tumor models with confirmed tumor-internal colonization (48 h) and monitored intra-tumor microbial dynamics through in vivo imaging. Compared to untreated controls, mice receiving kanamycin injections exhibited a marked reduction in tumor-internal bioluminescence signals as early as 24 h post-administration, accompanied by a narrowed microbial distribution range and significantly diminished peak signals (Fig. 5d). This phenomenon indicates that systemically administered kanamycin can penetrate tumor tissue via the bloodstream and inhibit the proliferation of engineered bacteria within the tumor microenvironment.

To further validate the imaging observations, tumor tissue homogenates were prepared and subjected to plate-counting assays. Results demonstrated a significant reduction in the number of cultivable colonies within tumors in the kanamycin-treated group compared to controls, although viable bacteria remained detectable (Fig. 5e). This indicates that at the administered dose, the antibiotic did not achieve complete eradication of the engineered bacteria but instead induced a state of “partial lysis and partial survival”. This outcome aligns with our MIC-based projections: the selected dosage effectively reduces microbial peak density within tumors while preserving sufficient viable bacteria to establish conditions for potential subsequent re-expansion or periodic release.

To evaluate the biosafety of the combined therapeutic strategy, peripheral blood samples were collected from treated mice following engineered bacterial administration and antibiotic-induced lysis. Hematological and biochemical analyses showed that key inflammatory indicators, including white blood cells, lymphocytes, neutrophils, monocytes, as well as the NLR and MLR ratios, remained within normal ranges, with no significant differences compared with the control group (Supplementary Fig. 4a–f). In addition, liver function markers, including ALT, AST, ALP, ALB, and GLB, as well as the renal function marker BUN, showed no evident alterations compared with controls, indicating no detectable hepatic or renal toxicity (Supplementary Fig. 4g–l).

Furthermore, histological analysis of major organs, including the heart, liver, spleen, lung, and kidney, revealed no obvious pathological abnormalities. Consistently, immunostaining for the inflammatory cytokine IL-6 showed no marked increase across these tissues compared with the control group (Supplementary Fig. 4m), suggesting the absence of systemic inflammatory responses. Together, these results support a favorable biosafety profile of the combined therapeutic strategy in vivo.

### Tumor-targeted aptamers release CXCL9 to exert antitumor effects in mice

**Fig. 6:**
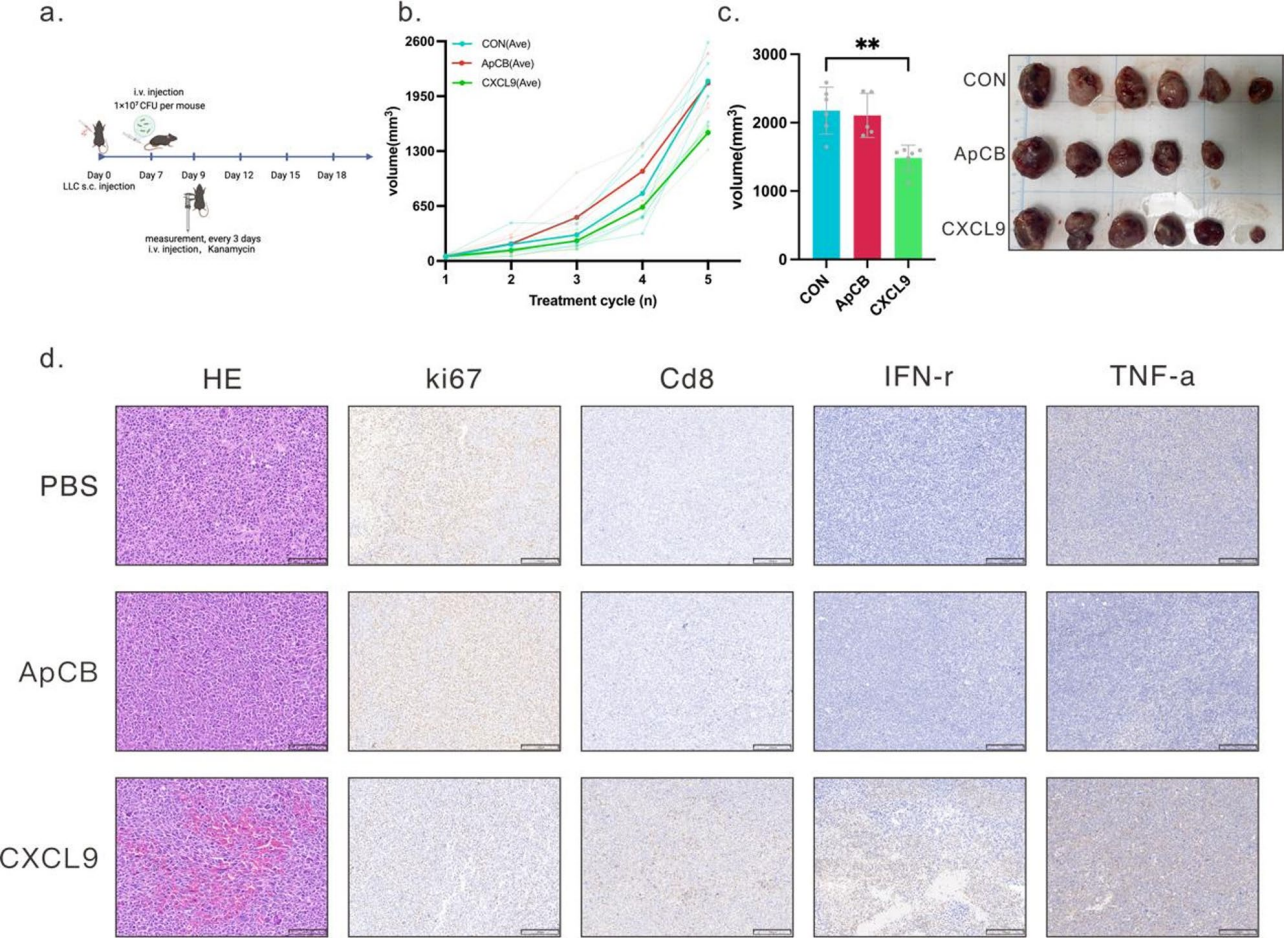
In vivo therapeutic effects of ApCBCXCL9. (**a**) Schematic of LLC tumor-bearing mice treatment. Created with BioRender.com. The mice were randomly assigned into three groups following inoculation: the PBS group, the ApCB group, and the ApCB_CXCL9_ group. ApCB group: Mice treated with aptamer-conjugated engineered bacteria (ApCB) lacking CXCL9 expression, serving as a bacterial vector control. ApCB_CXCL9_ group: Mice treated with aptamer-conjugated engineered bacteria engineered to express CXCL9, used to evaluate the therapeutic effect of CXCL9 delivery. (**b**) Individual growth curves and mean growth curves of LLC tumor volume in each group of mice, *n* = 6. (**c**) Representative photographs of tumor tissue extracted from mice at the end of treatment. Scale bar: 1 cm. (**d**) Representative H&E-stained sections and immunohistochemical staining images for Ki67, CD8, IFN-γ, and TNF-α in tumors following different treatments. Images depict representative results from three independent tumor tissue samples. Scale bar: 100 μm

Based on the aforementioned in vivo targeting data, we further evaluated the antitumor efficacy of aptamer-modified engineered bacteria expressing CXCL9 in LLC tumor-bearing mice. Mice were randomly assigned to three groups: a PBS control group, an EcN group without CXCL9 expression, and a CXCL9 treatment group expressing CXCL9. Both the EcN and CXCL9 groups were aptamer-modified. Building upon prior observations that engineered bacteria achieve stable enrichment within tumor regions within 48 h post-administration, we employed a biennial antibiotic administration strategy to maintain controlled bacterial persistence within tumors while minimizing systemic exposure. Tumor tissues were harvested and analyzed at the predetermined endpoint (Fig. 6a).

Tumor growth curves demonstrated markedly reduced tumor volumes in CXCL9-engineered bacteria-treated mice compared to controls (Fig. 6b-c). HE staining revealed extensive areas of necrosis within tumors in this group, consistent with enhanced anti-tumor activity. Immunohistochemistry further showed a significant decrease in Ki67-positive proportion, indicating suppressed tumor proliferation (Fig. 6d).

Regarding immune infiltration, CD8⁺ T cell density markedly increased in the CXCL9 group (Fig. 6d). Their effector function markers (including IFN-γ and TNF-α) also exhibited pronounced enhancement (Fig. 6d), suggesting that CXCL9 release by engineered bacteria may effectively improve intratumoral T cell recruitment and activation, thereby promoting antitumor immune responses. Overall, this therapeutic strategy demonstrates potential for promoting immune infiltration and inhibiting tumor progression while maintaining systemic controllability.

## Discussion

This study aims to address one of the critical bottlenecks in solid tumor immunotherapy—persistent T-cell exhaustion and the resulting inadequate immune infiltration [41]. By constructing CXCL9-secreting engineered bacteria, we establish a sustained intratumoral CXCL9 chemotactic gradient within the tumor microenvironment (TME). This promotes the migration of effector T cells into the tumor interior and ameliorates the local immunosuppressive state. Compared to traditional delivery strategies based on recombinant proteins or viral vectors, bacterial vectors offer unique advantages as platforms for delivering immunomodulatory molecules due to their hypoxia-dependent proliferation [42], high expression capacity, and programmable controllability. Our findings further validate the potential advantages of engineered bacteria in regulating tumor-infiltrating T cells and reveal their broader role in remodeling the immune microenvironment.

Firstly, our data demonstrate that sustained intratumoral expression of CXCL9 significantly enhances CD8⁺ T cell infiltration, accompanied by partial reversal of T cell exhaustion phenotypes. This aligns with prior evidence regarding the critical role of the CXCL9/10–CXCR3 axis in regulating anti-tumor immune migration pathways. Traditional approaches struggle to maintain stable gradients in solid tumors due to short half-lives [43], extensive tissue distribution, and limited local delivery efficiency. Sustained local expression achieved through engineered bacteria effectively resolves this challenge. The findings from this study provide a novel, viable strategy for enhancing ICB response rates.

Secondly, we combined tumor-targeting aptamers with engineered bacteria to enhance their colonization efficiency within tumors [44]. This study provides extended validation of the targeting capability of the AS1411 aptamer within a mouse lung adenocarcinoma model [45], thereby significantly broadening its application scope. This not only furnishes fresh evidence for the aptamer’s cross-system compatibility but also demonstrates that the “aptamer-microbial carrier” combination strategy holds promise as a robust direction for future precision immunotherapy delivery systems.

Although the system we constructed demonstrated significant efficacy in immune enhancement, this study retains several limitations. Firstly, sustained expression of CXCL9 may induce robust immune activation or result in tissue toxicity; future work requires establishing more stringent controllable expression systems, such as employing logic gates, immune-stimulation response elements, or small-molecule–responsive control switches. Secondly, the targeting efficacy of aptamer-mediated delivery requires further evaluation in models exhibiting greater heterogeneity [46], such as spontaneous lung adenocarcinoma models, PD models, or settings with pronounced immune disruption. Moreover, employing antibiotics to assist engineered bacteria in drug release and controlling their in vivo clearance is not an optimal strategy [47]. Subsequent research should focus on developing lysis protocols based on the engineered bacteria’s intrinsic properties, such as those triggered by tumor hypoxic environments or weakly acidic conditions. The host immune system’s clearance of engineered bacteria may still compromise their stability and persistence, necessitating further systematic validation of these factors during the preclinical phase [48].

It is noteworthy that our immunofluorescence analysis revealed a localized enrichment of CD86⁺ macrophages following bacterial colonization within tumors, suggesting a shift toward a more pro-inflammatory phenotype [49]. In line with this observation, flow cytometric analysis further demonstrated a reduction in CD206⁺ immunosuppressive M2-like macrophages, supporting a reprogramming of tumor-associated macrophages toward a less immunosuppressive state. This effect may be associated with the release of lipopolysaccharide (LPS) from the engineered bacteria, which is known to modulate macrophage polarization. Although this study did not further elucidate the underlying signal axes (such as TLR4–NF-κB activation, fatty acid metabolism remodeling, or altered myeloid cell energy metabolism), this phenomenon suggests engineered bacteria may concurrently possess innate immune reprogramming potential, thereby synergistically alleviating immune suppression within the tumor microenvironment through multi-level regulation. This unexpected yet instructive discovery offers novel directions for designing future combined engineered bacterial interventions that “enhance T-cell immunity & remodel myeloid immunity”.

Collectively, this study demonstrates a novel tumor-immunity-enhancing strategy based on engineered bacteria delivering CXCL9, further enhanced by aptamers for improved tumor targeting. We have not only validated the efficacy of this delivery approach in ameliorating T-cell exhaustion and enhancing immune infiltration, but also revealed the potential additional effect of engineered bacteria in remodeling the TME through innate immune regulation. Future integration with immune checkpoint inhibitors, myeloid immune modulators, metabolic intervention drugs, or other synthetic biology control circuits holds promise for further amplifying the anti-tumor effects of this strategy, offering a novel avenue for overcoming bottlenecks in solid tumor immunotherapy [50, 51].

## Materials and methods

### Materials and bacterial strains

1-Ethyl-3-(3-dimethylaminopropyl) carbodiimide hydrochloride (EDC) and N-hydroxysuccinimide (NHS) were obtained from Adamas-beta (Shanghai, China).The AS1411 aptamer (5ʹ-GGT GGT GGT GGT TGT GGT GGT GGT GGT TTT TTT TTT TT-NH2-3ʹ) was synthesized by Sangon Biotech (Shanghai, China).E. coli Nissle 1917 (EcN) was purchased from the General Microbial Culture Collection Center (GMCC, China).The murine lung adenocarcinoma cell line LLC was obtained from Haixing Bio (Suzhou, China). LLC cells were maintained in DMEM-H medium (BC-M-005-500, Bio-Channel, Nanjing, China) supplemented with 10% fetal bovine serum (FBS, Umedium, Hefei, China) and 1% penicillin/streptomycin (BC-CE-007,Bio-Channel).Cells were cultured at 37 °C in a humidified incubator containing 5% CO_2_,and routinely tested for mycoplasma contamination. Plasmids pRSF-J23119-pelB-CXCL9-his, pRSF-J23119-pelB-mCherry-his, and pGEN-luxCDABE were purchased from Tsingke (Chuzhou, China) and used as received.

### Animals

All animal experiments were conducted in accordance with protocols approved by the Ethics Committee of Xuzhou Medical University. Male C57BL/6J mice (6–8 weeks of age,18–20 g) were purchased from GemPharmatech Co.,Ltd.(Nanjing, China). Animals were housed under specific pathogen–free (SPF) conditions with a 12 h light/dark cycle, controlled temperature (25 °C), and relative humidity (55%), with free access to food and water. All procedures complied with the institutional guidelines for the care and use of laboratory animals at Xuzhou Medical University. Mice were euthanized when tumor volume exceeded 2000 mm³, in accordance with predefined humane endpoints, and no animal exceeded the maximum allowable tumor burden.

### Characterization

Fluorescence imaging of cells was performed using a confocal laser scanning microscope (Olympus Corporation, Japan).Flow cytometric analysis was conducted on a BD LSRII system (BD Biosciences, USA) to quantitatively assess cellular fluorescence. In vivo imaging of tumor-bearing mice was carried out using an IVIS Lumina III imaging system (PerkinElmer, USA). Morphological characterization was performed using scanning electron microscopy (SEM).

### Bacterial growth

EcN strains harboring the plasmids pRSF-J23119-pelB-CXCL9-his or pGEN-luxCDABE were cultured overnight in LB medium supplemented with the appropriate antibiotic selection. Overnight cultures were subsequently diluted into fresh LB medium and incubated under standard bacterial growth conditions. Bacterial cells were collected, washed in PBS, and resuspended for downstream applications. Bacterial concentrations were estimated by serial dilution and plating onto LB agar, followed by overnight incubation to determine colony-forming units (CFU).

### Aptamer-conjugated bacteria (ApCB)

Amino-functionalized aptamers were conjugated to the bacterial surface through EDC/NHS-mediated amide coupling. EcN expressing the mCherry reporter (EcN_mCherry_) was suspended in PBS, followed by the addition of amino-modified aptamers together with EDC and NHS at predetermined ratios. During the coupling reaction, EDC activates carboxyl groups to form O-acylisourea intermediates, whereas NHS stabilizes the activated species, thereby enhancing the efficiency of amide bond formation. After incubation, the bacterial suspension was washed to remove unreacted components and subsequently analyzed by flow cytometry (BD LSRII). Data were processed using FlowJo software (TreeStar).

### Binding of ApCB to cancer cells

LLC cells were seeded into confocal culture dishes and maintained in DMEM-H supplemented with fetal bovine serum and antibiotics under standard incubator conditions. After adherence, the culture medium was replaced with fresh DMEM-H, and cells were incubated with either EcN or ApCB preparations for a defined period to allow bacteria–cell interactions. Following incubation, cells were washed with PBS to remove unbound bacteria and subsequently imaged using a confocal laser scanning microscope to visualize bacterial association. For quantitative assessment of ApCB–cell binding, LLC cells were harvested and incubated with fluorescently labeled EcN_mCherry_ or ApCB_mCherry_. Cell-associated fluorescence was evaluated using a BD LSRII flow cytometer, and data were analyzed with FlowJo software (TreeStar).

### Scanning electron microscopy

To directly visualize the interaction between bacteria and cancer cells, samples were chemically fixed, washed thoroughly to remove residual fixative, and subjected to graded dehydration using ethanol solutions. The dehydrated specimens were subsequently transitioned through an intermediate solvent and dried using a CO_2_ critical point dryer to preserve structural integrity. Dried samples were sputter-coated with a thin layer of gold to enhance conductivity and imaged using a scanning electron microscope.

### Subcutaneous tumor model

Subcutaneous tumor models were established in male C57BL/6J mice (6–8 weeks of age), which were maintained under specific pathogen–free (SPF) conditions. For the LLC tumor model, LLC cells suspended in serum-free medium were injected into the right axillary region of each mouse to establish palpable tumors. Tumor growth was monitored using digital calipers, and tumor volume was calculated using the standard ellipsoid formula: volume=(width^2^×length×0.5).

### Bacterial administration via tail vein, tumor colonization, and biodistribution

To investigate the in vivo biodistribution of E. coli Nissle 1917 (EcN) and 5ApCB, a Lewis lung carcinoma (LLC) tumor-bearing mouse model was established. Briefly,1 × 10^6^ LLC cells in 100 µL of serum-free DMEM were injected subcutaneously into the right axilla of each C57BL/6J mouse. When tumor volumes reached approximately 200 mm^3^, mice were randomly assigned to three groups: PBS, EcN_LuxCDABE_, and 5ApCB_LuxCDABE_(*n* = 3 per group). To assess bacterial accumulation in tumors, tumor-bearing mice were administered 1 × 10^7^ CFU of LuxCDABE-expressing EcN or 5ApCB via tail vein injection. In vivo imaging was performed at 12, 36 and 60 h post-injection using a live animal imaging system. At the indicated time points, mice were euthanized for tissue collection, including heart, liver, spleen, lung, kidney, and tumor, which were subsequently imaged ex vivo. All images were analyzed using the IVIS Lumina III system. For each group, a region of interest (ROI) was defined for the tumor tissue. The mean intensity within the tumor ROI was quantified, and the corresponding background intensity was subtracted for each frame.

### Quantification of bacterial biodistribution

Following in vivo and ex vivo imaging, major organs—including the heart, liver, spleen, lungs, kidneys, and tumor tissues—were harvested and mechanically homogenized. Homogenates were serially diluted in LB medium and plated onto LB agar supplemented with appropriate antibiotics to selectively culture the engineered bacterial strains. After incubation under standard bacterial growth conditions, colony-forming units (CFU) were quantified. ImageJ software was used to analyze bacterial colonies on agar plates.

### Colony PCR and agarose gel electrophoresis

Colony PCR was performed by directly adding a small amount of bacterial culture to the PCR reaction mixture, with an extended initial denaturation step to lyse the cells and release genomic material, eliminating the need for prior DNA purification. PCR amplification was carried out using vector- or insert-specific primers in a conventional reaction system containing template, primers, buffer, and DNA polymerase. Thermal cycling conditions followed standard protocols appropriate for the selected primer set. PCR products were subsequently resolved by agarose gel electrophoresis using gels prepared in a conventional electrophoresis buffer system. Samples mixed with loading buffer were loaded onto the gel and separated under standard electrophoretic conditions until adequate band separation was achieved. DNA bands were visualized using a UV or blue-light imaging system following staining with a commercially available nucleic acid dye.

### Western blot analysis

EcN strains expressing CXCL9 (EcN_CXCL9_) were cultured under selective conditions, and bacterial supernatants were collected for protein analysis. Samples were mixed with loading buffer and heat-denatured prior to electrophoresis. Proteins were separated by SDS–PAGE and subsequently transferred onto methanol-activated PVDF membranes using standard transfer conditions. Membranes were blocked and incubated with primary antibodies against CXCL9 and β-tubulin at optimized dilutions, followed by incubation with HRP-conjugated secondary antibodies. After thorough washing, immunoreactive bands were visualized using an enhanced chemiluminescence (ECL) detection system, and signals were captured with a digital imaging instrument.

### ELISA analysis

EcN cultures grown under standard conditions were used as controls, and bacterial supernatants and cell pellets were collected during the logarithmic growth phase for analysis. Similarly, EcN strains engineered to express CXCL9 (EcN_CXCL9_) were cultured under selective conditions, and supernatants and pellets were harvested at the corresponding growth stage. Following sample preparation, aliquots of culture supernatants were subjected to quantitative analysis using a commercial mouse CXCL9 ELISA kit (KE10067, Proteintech, Wuhan, China) according to the manufacturer’s instructions to evaluate the biological activity of CXCL9 produced by EcN_CXCL9_.Absorbance was measured at 450 nm.

### Transwell migration assay

CD3⁺ T cells were isolated from the spleens of C57BL/6J mice using a magnetic bead–based negative selection kit (EasySep™ Mouse T Cell Isolation Kit, STEMCELL Technologies). Purified T cells were seeded into the upper chambers of Transwell inserts, while the lower chambers were supplemented with recombinant CXCL9 protein and/or LLC cells to establish a chemotactic gradient. Following incubation under standard culture conditions, migrated cells in the lower chambers were collected and stained with fluorophore-conjugated antibodies against CD3, CD4 and CD8, with fluorescence-minus-one (FMO) controls included for gating precision. Flow cytometric acquisition was performed on a BD LSRII cytometer, and data were analyzed using FlowJo software (TreeStar).

### Chemokine-mediated immune cell recruitment and histological analysis

LLC tumor–bearing mouse models were established by subcutaneous inoculation of LLC cells into the right axillary region of C57BL/6J mice. Once tumors reached a predetermined size, mice were randomly divided into two groups to receive either PBS or recombinant CXCL9 via intratumoral administration at scheduled intervals. Tumor growth was monitored longitudinally using digital calipers, and tumor tissues were harvested at the experimental endpoint for immunological and histological assessment. Collected tumor samples were fixed, embedded in paraffin, and processed for immunohistochemical staining.CD8⁺ T-cell infiltration and CXCL9 expression within the tumor microenvironment were evaluated to determine chemokine-mediated immunomodulatory effects.

### Intravenous antibiotic intervention for modulating intratumoral bacterial density and safety evaluation

LLC tumor–bearing mouse models were established to investigate the effect of systemic antibiotic administration on modulating intratumoral bacterial burden and treatment-associated tissue responses. Once tumors reached a predetermined volume, mice were randomized into three groups: PBS, ApCB_LuxCDABE_ and ApCB_LuxCDABE_ combined with systemic antibiotic treatment. Following intravenous administration of bacteria and subsequent confirmation of tumor colonization, the antibiotic-treated group received intravenous antimicrobial intervention to selectively reduce bacterial density within the tumor microenvironment. Longitudinal monitoring was performed at predefined intervals using an in vivo imaging system to assess bacterial distribution and quantify intratumoral bacterial load before and after antibiotic treatment. Tumors collected at the endpoint were fixed, embedded in paraffin, and processed for immunofluorescence staining to evaluate tissue architecture and bacterial localization across treatment groups.

### Bacteria-mediated cytokine release for cancer therapy and histological analysis

LLC tumor–bearing mouse models were established by subcutaneous inoculation of LLC cells into the right axillary region of C57BL/6J mice. When tumors reached a predefined volume, mice were randomly divided into three groups and intravenously administered PBS, EcN, or ApCB at scheduled treatment intervals to evaluate the antitumor effects mediated by bacteria-induced cytokine release. Tumor growth was monitored periodically using digital calipers. For histopathological analysis, tumor tissues were collected at the experimental endpoint, fixed in paraformaldehyde, embedded in paraffin, and subjected to hematoxylin and eosin (H&E) staining to evaluate tumor morphology. Immunohistochemical staining for Ki-67, CD8⁺ T cells, IFN-γ and TNF-α was performed to assess cell proliferation and immune activation within the tumor microenvironment across treatment groups.

### Statistical analysis

All experiments were performed at least in triplicate. Data are presented as mean ± standard deviation (SD). The exact sample size for each experimental group is indicated in the corresponding figure legends. Statistical comparisons between two groups were performed using Student’s t-test, whereas comparisons among three or more groups were analyzed using one-way analysis of variance (ANOVA) followed by appropriate post hoc tests. Statistical analyses were conducted using SPSS software (version 17.0). A p-value < 0.05 was considered statistically significant. Significance levels are denoted as follows: ns, not significant; *p* < 0.05;*p < 0.01;**p < 0.001.

## Electronic Supplementary Material

Below is the link to the electronic supplementary material.

Supplementary Material 1

Supplementary Material 2

Supplementary Material 3

Supplementary Material 4

Supplementary Material 5

## Data Availability

The datasets used and/or analyzed during the current study are available from the corresponding author on reasonable request.

## Abbreviations

TME: Immunosuppressive tumor microenvironment
ApCB: Aptamer-conjugated engineered bacterial strain
MIC: Minimum inhibitory concentration
ICB: Immune checkpoint blockade
ORR: Objective response rate
NSCLC: Non-small cell lung cancer
RCC: Renal cell carcinoma
TME: Tumor microenvironment
LLC: Lewis lung carcinoma
Tpex: Precursor exhausted T cells
Tex: Terminally exhausted T cells
EcN: E. coli Nissle 1917
TAMs: Tumor-associated macrophages
TCM: Tumor-conditioned medium
