# Supplementary figures and images for: Tumor-targeted aptamer-conjugated engineered bacteria for CXCL9 cytokine delivery in non-small cell lung cancer immunotherapy
Source: J Transl Med. 2026 Apr 27;24:590. doi: 10.1186/s12967-026-08194-y (PMC13123157; doi:10.1186/s12967-026-08194-y)

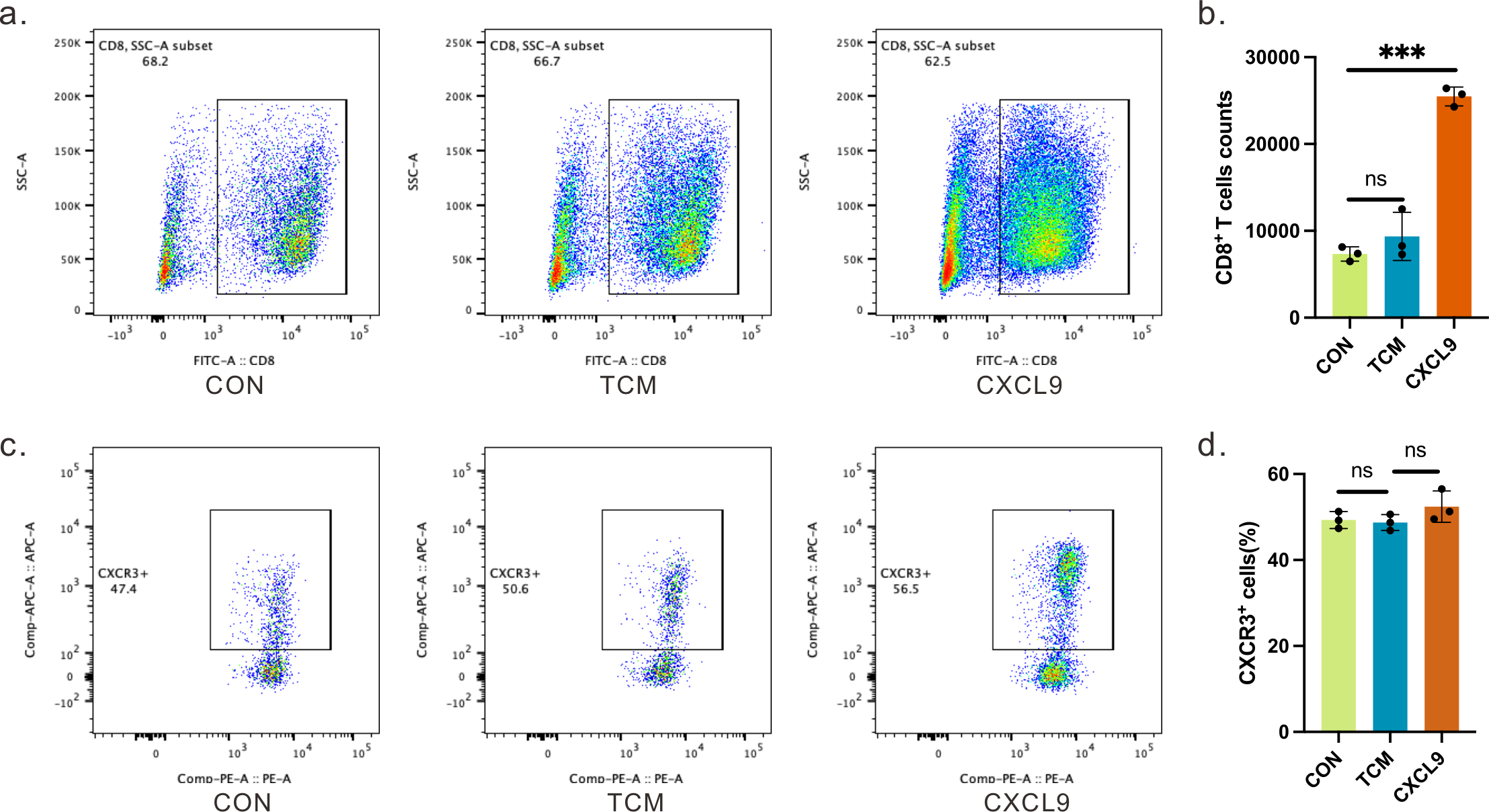

Supplement: Supplementary file 1 — Supplementary Material 1 [file 12967_2026_8194_MOESM1_ESM.tif]

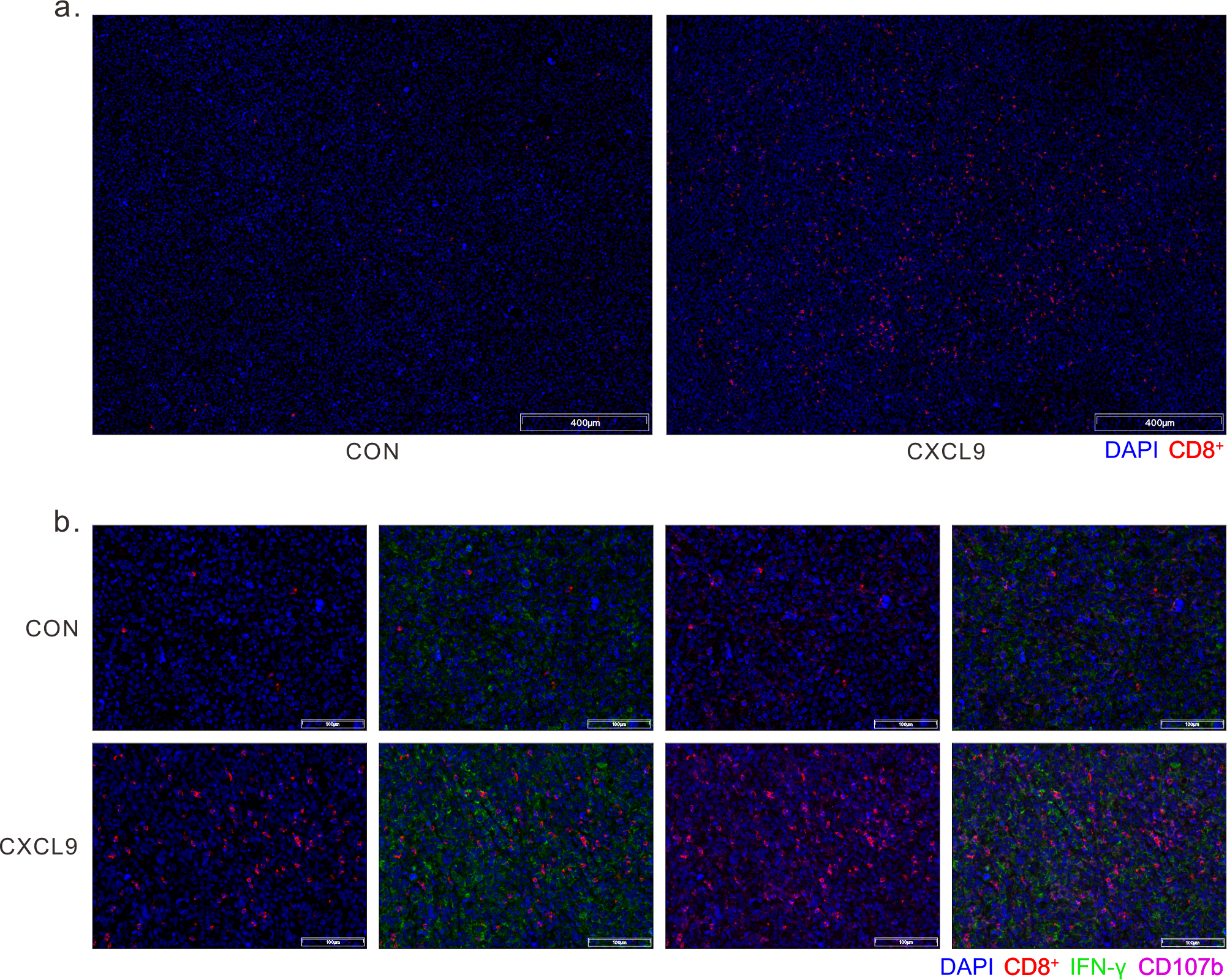

Supplement: Supplementary file 2 — Supplementary Material 2 [file 12967_2026_8194_MOESM2_ESM.tif]

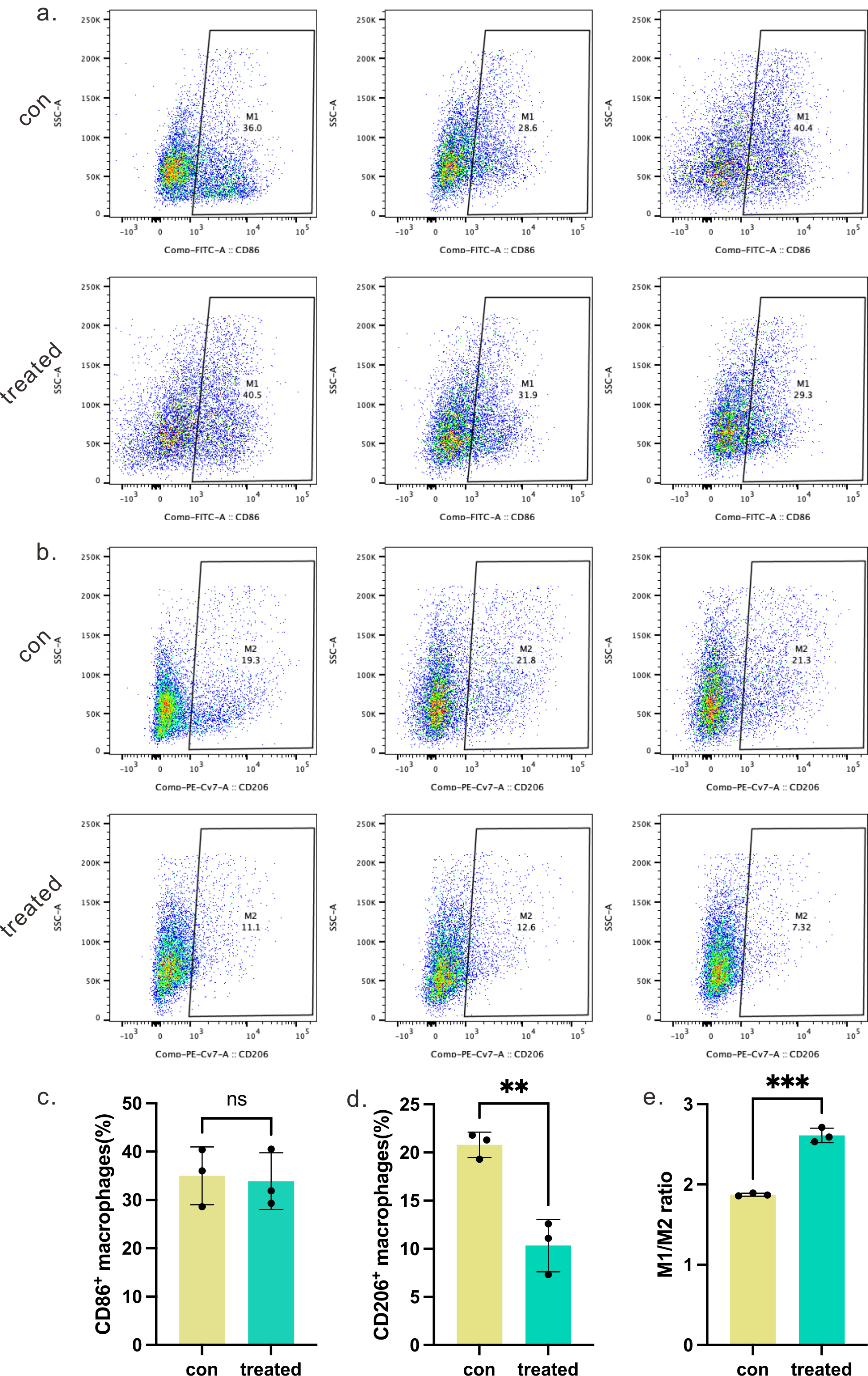

Supplement: Supplementary file 3 — Supplementary Material 3 [file 12967_2026_8194_MOESM3_ESM.tif]

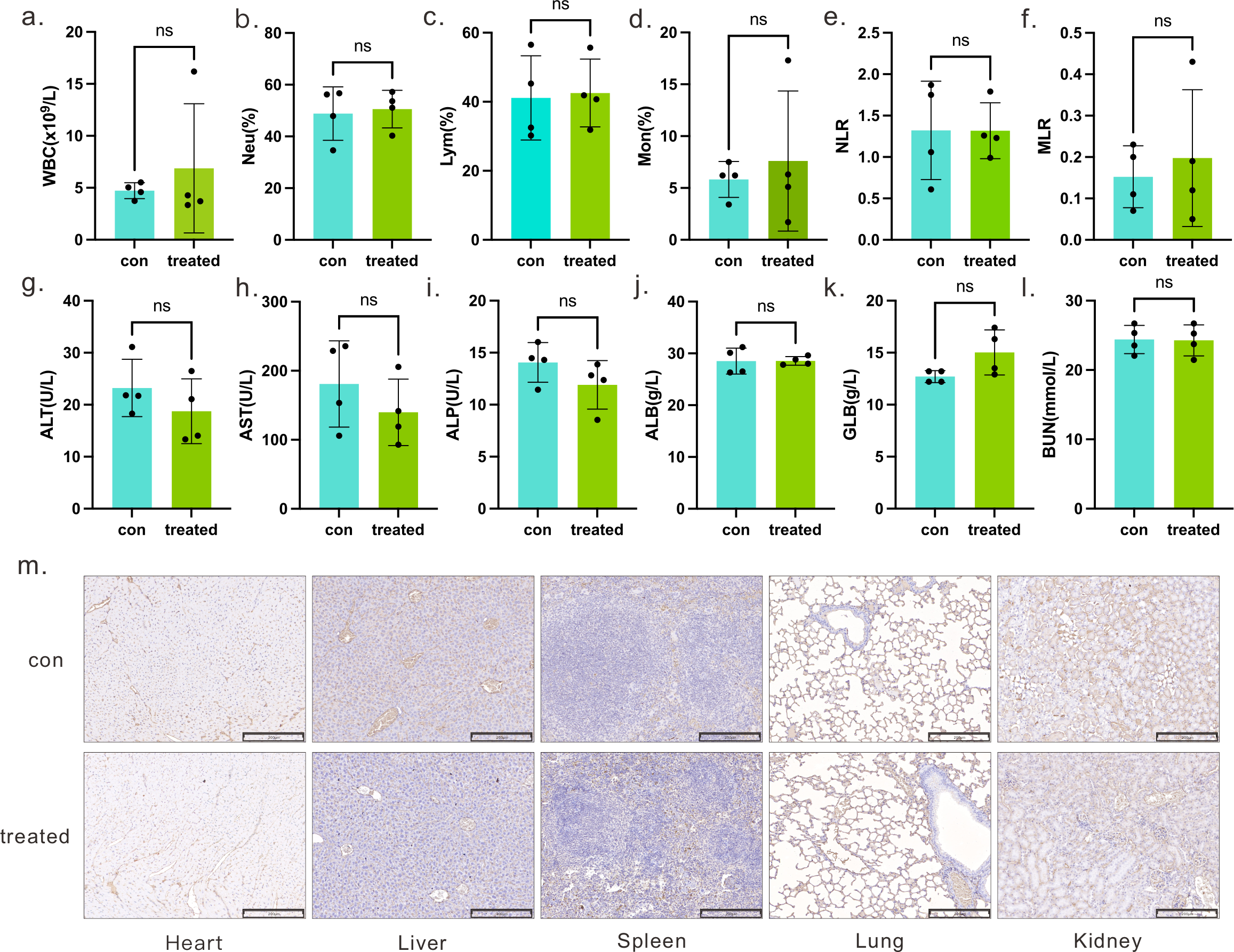

Supplement: Supplementary file 4 — Supplementary Material 4 [file 12967_2026_8194_MOESM4_ESM.tif]
