# Supplementary material for: Tumor-targeted aptamer-conjugated engineered bacteria for CXCL9 cytokine delivery in non-small cell lung cancer immunotherapy
Source: J Transl Med. 2026 Apr 27;24:590. doi: 10.1186/s12967-026-08194-y (PMC13123157; doi:10.1186/s12967-026-08194-y)
Supplement: Supplementary file 5 — Supplementary Material 5 [file 12967_2026_8194_MOESM5_ESM.docx]

**Supplementary Figures：**

**
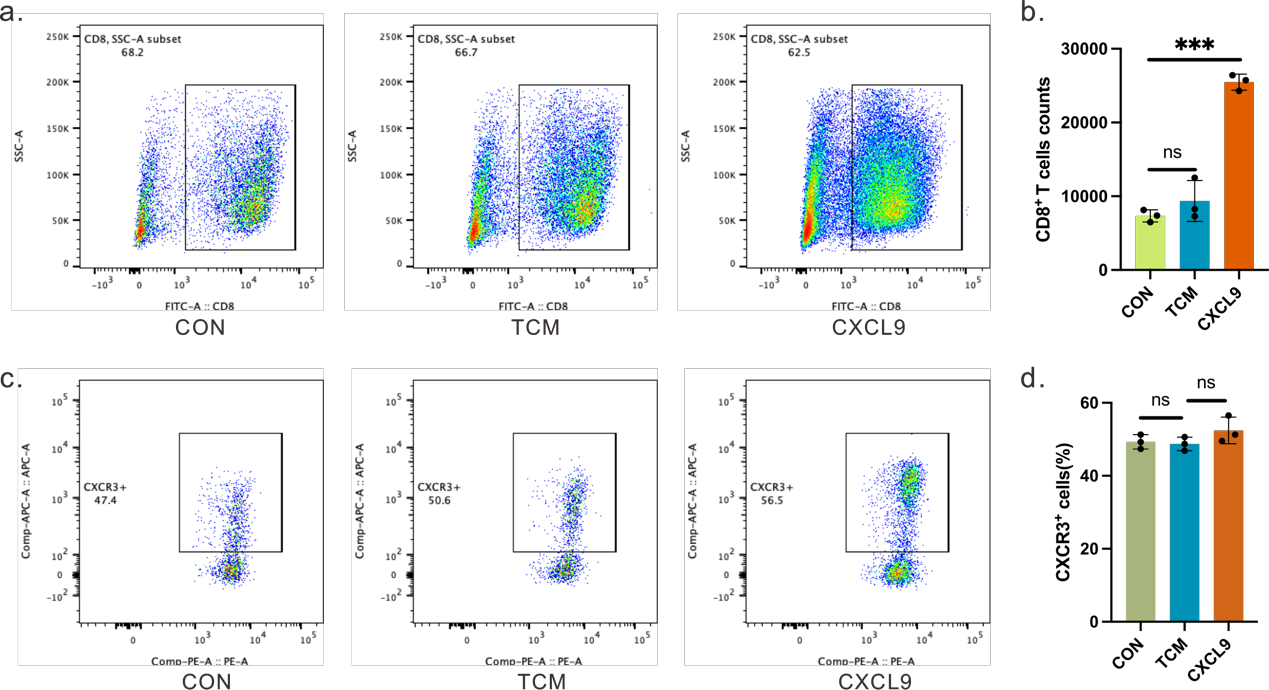
**

**Supplementary Figure 1**

**a-d**. Transwell migration assay. CD3⁺ T cells (1 × 10^6^cells per well) isolated from the spleens of C57BL/6J mice were seeded in the upper chamber. The lower chamber contained tumor cell–conditioned medium or purified CXCL9 protein to assess chemotactic migration. **a, c.** After 24 h incubation, cells that migrated to the lower chamber were collected and stained with fluorophore-conjugated anti-CD3 and anti-CD8 antibodies. Flow cytometry was used to quantify the proportion of CD8⁺ T cells within the CD3⁺ population, as well as the proportion of CXCR3⁺ cells within CD8⁺ T cells. Representative results from three independent samples are shown. **b, d.** Quantification of CD8⁺ T cells and CXCR3⁺ T cells obtained by flow cytometry across different groups. Data are presented as mean ± SD. Statistical significance was determined using a two-tailed Student’s t-test. ns, not significant.

**
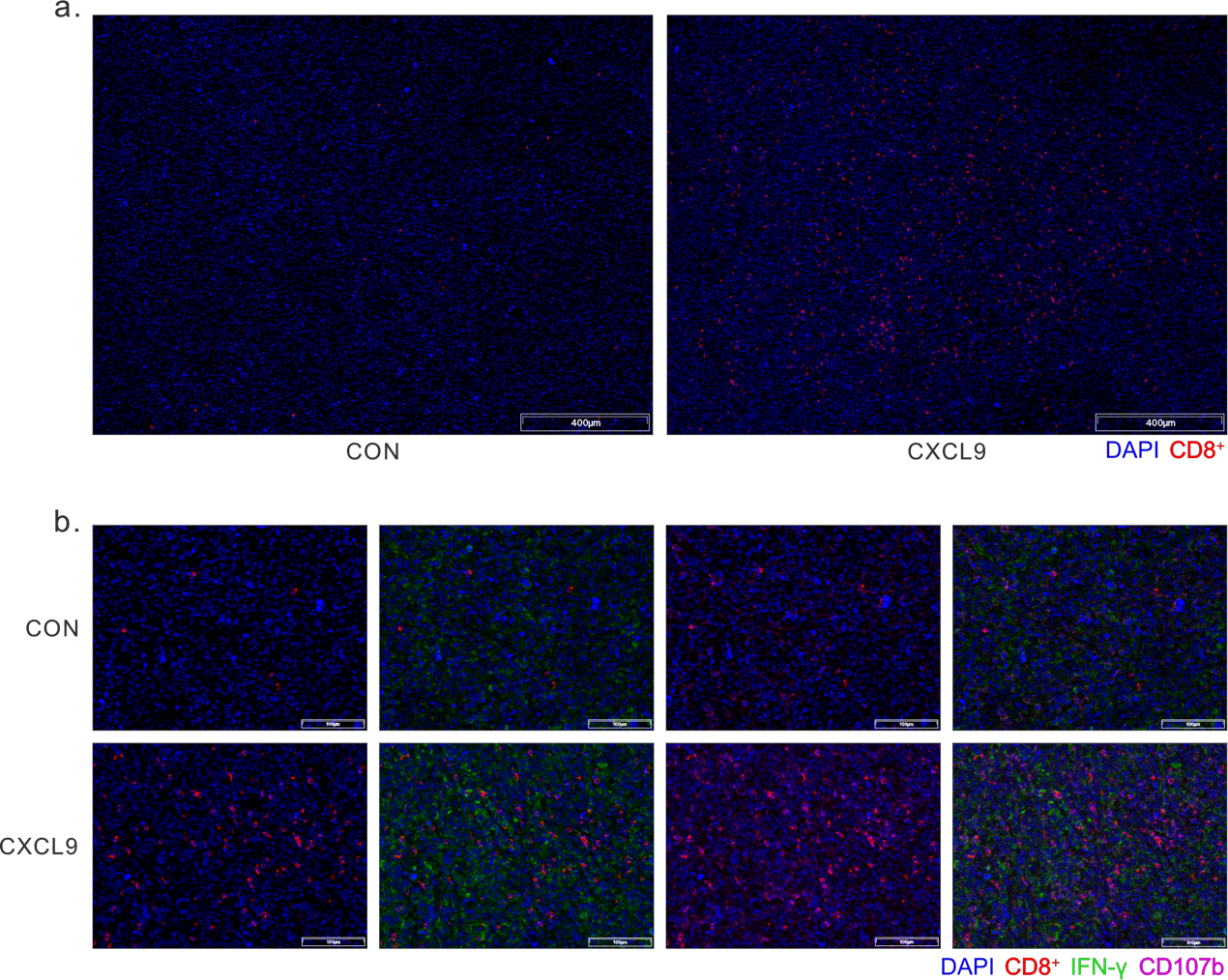
**

**Supplementary Figure 2**

**a-b**. Immunofluorescence staining of tumor sections from control and CXCL9-treated groups, showing CD8⁺ T cells, CD107b, and IFN-γ expression. Representative images from three independent tumor samples per group are shown. Scale bars: 400 μm (a), 100 μm (b).

**
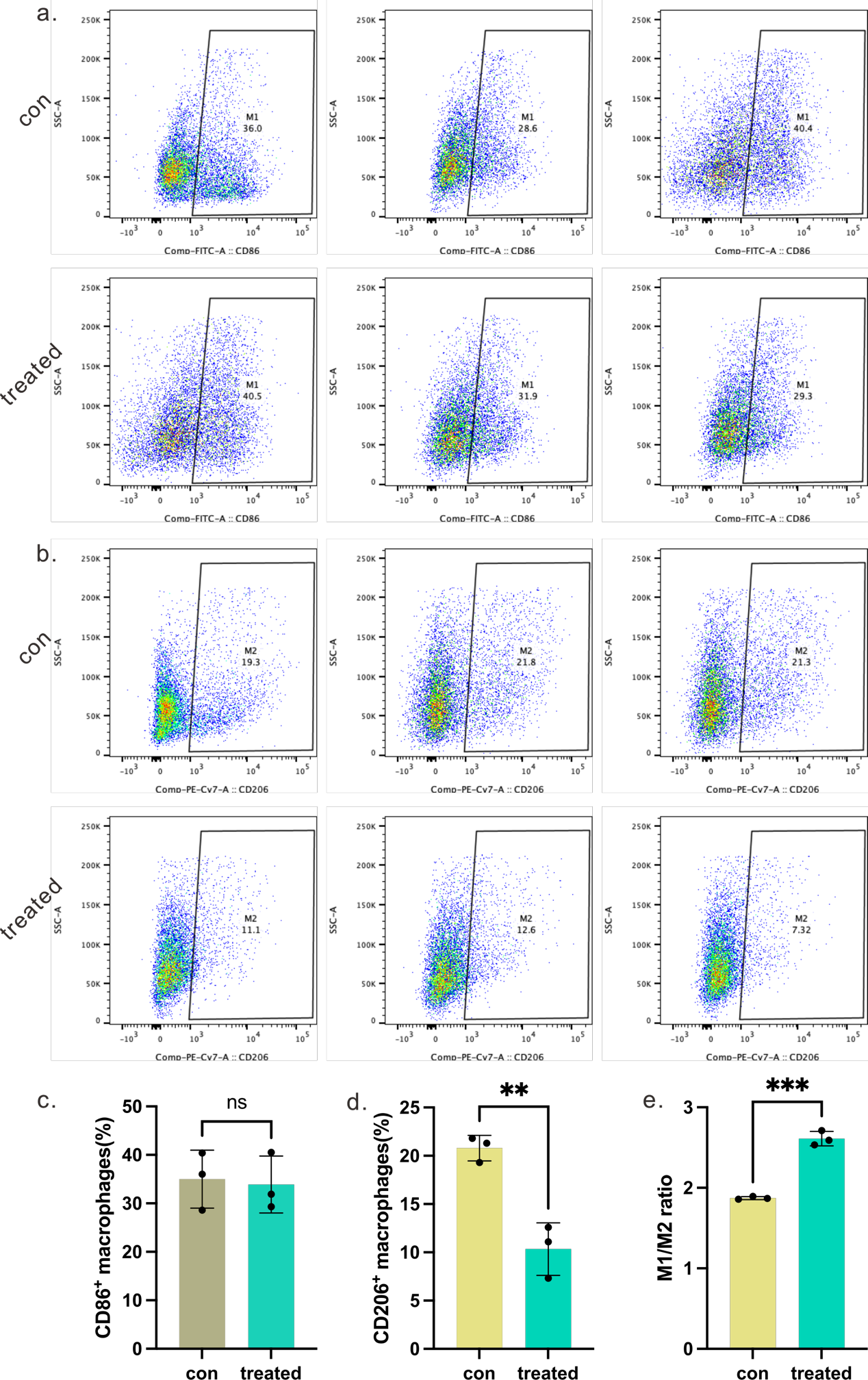
**

**Supplementary Figure 3**

**a.** Tumor tissues from PBS and 5ApCBLux-treated mice were dissociated and subjected to red blood cell lysis, followed by staining with fluorophore-conjugated antibodies against CD11b, CD86, and CD206. Flow cytometry was performed to identify macrophage subsets (M1 and M2) and quantify their relative proportions. Representative results from three independent samples are shown. **b-e.** Quantitative analysis of macrophage polarization, including the proportion of M1 macrophages within total macrophages (b), the proportion of M2 macrophages (c), and the M1/M2 ratio (e). Data are presented as mean ± SD. Statistical significance was determined using a two-tailed Student’s t-test. ns, not significant.

**
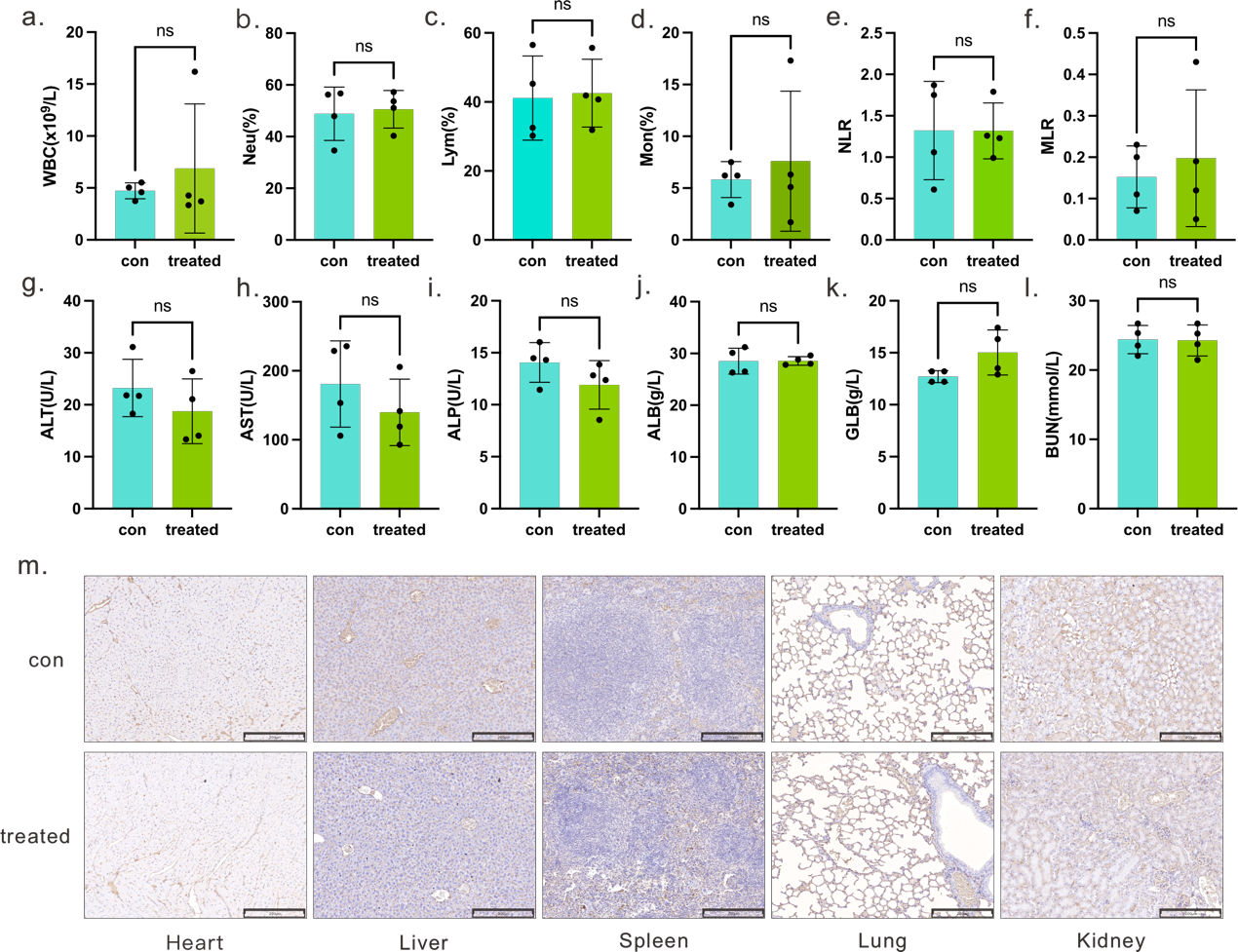
**

**Supplementary Figure 4**

**a-f.** Hematological analysis. Peripheral blood parameters were measured to evaluate systemic inflammatory responses. Comparisons between untreated control mice and mice receiving combined bacterial and antibiotic treatment included white blood cells (WBC), neutrophils (Neu), lymphocytes (Lym), monocytes (Mon), neutrophil-to-lymphocyte ratio (NLR), and monocyte-to-lymphocyte ratio (MLR). No significant differences were observed between groups, indicating that combined bacterial and antibiotic treatment does not affect blood composition. **g-l.** Serum biochemical analysis. Key indicators of organ function were assessed, including alanine aminotransferase (ALT), aspartate aminotransferase (AST), alkaline phosphatase (ALP), albumin (ALB), globulin (GLB), and blood urea nitrogen (BUN). No significant differences were detected between groups, indicating preserved organ function following combined bacterial and antibiotic treatment. **m.** Immunohistochemical staining of IL-6 in heart, liver, spleen, lung, and kidney sections from control and 5ApCBLux-treated mice. Representative images from three independent samples per organ are shown. Scale bar: 200 μm. No apparent differences were observed between groups, further supporting the biosafety of the treatment.
